# Characterization of the Promoter, MxiE Box and 5′ UTR of Genes Controlled by the Activity of the Type III Secretion Apparatus in *Shigella flexneri*

**DOI:** 10.1371/journal.pone.0032862

**Published:** 2012-03-12

**Authors:** Clotilde Bongrand, Philippe J. Sansonetti, Claude Parsot

**Affiliations:** 1 Unité de Pathogénie Microbienne Moléculaire, Institut Pasteur, Paris, France; 2 INSERM U786, Paris, France; 3 Université Paris Diderot, Sorbonne Paris Cité (Cellule Pasteur), Paris, France; Wadsworth Center, New York State Dept. Health, United States of America

## Abstract

Activation of the type III secretion apparatus (T3SA) of *Shigella flexneri*, upon contact of the bacteria with host cells, and its deregulation, as in *ipaB* mutants, specifically increases transcription of a set of effector-encoding genes controlled by MxiE, an activator of the AraC family, and IpgC, the chaperone of the IpaB and IpaC translocators. Thirteen genes carried by the virulence plasmid (*ospB*, *ospC1*, *ospD2*, *ospD3*, *ospE1*, *ospE2*, *ospF*, *ospG*, *virA*, *ipaH1.4*, *ipaH4.5*, *ipaH7.8* and *ipaH9.8*) and five genes carried by the chromosome (*ipaHa-e*) are regulated by the T3SA activity. A conserved 17-bp MxiE box is present 5′ of most of these genes. To characterize the promoter activity of these MxiE box-containing regions, similar ∼67-bp DNA fragments encompassing the MxiE box of 14 MxiE-regulated genes were cloned 5′ of *lacZ* in a promoter probe plasmid; β-galactosidase activity detected in wild-type and *ipaB* strains harboring these plasmids indicated that most MxiE box-carrying regions contain a promoter regulated by the T3SA activity and that the relative strengths of these promoters cover an eight-fold range. The various MxiE boxes exhibiting up to three differences as compared to the MxiE box consensus sequence were introduced into the *ipaH9.8* promoter without affecting its activity, suggesting that they are equally efficient in promoter activation. In contrast, all nucleotides conserved among MxiE boxes were found to be involved in MxiE-dependent promoter activity. In addition, we present evidence that the 5′ UTRs of four MxiE-regulated genes enhance expression of the downstream gene, presumably by preventing degradation of the mRNA, and the 5′ UTRs of two other genes carry an ancillary promoter.

## Introduction

The type III secretion (T3S) pathway is used by numerous Gram-negative bacteria to deliver virulence proteins to the membrane or the cytoplasm of cells of their host, where they interfere with cellular signaling pathways. T3S systems comprise (i) a secretion apparatus (T3SA) spanning the bacterial envelope, (ii) translocators transiting through the T3SA that are inserted into the membrane of the host cell in which they form a pore, (iii) effectors injected into host cells via the T3SA and the translocator pore, (iv) molecular chaperones associating with translocators and some effectors within the bacterial cytoplasm, (iv) and transcription regulators [1], [2]. In addition to resulting in the transit of translocators and effectors, activation of the T3SA upon contact of bacteria with host cells activates transcription of either most genes of the T3S system or a subset of effector-encoding genes. Mechanisms by which the T3SA activity controls gene transcription involve mutually exclusive interactions between transcription activators, chaperones and T3SA substrates [3], [4].

Members of the genus *Shigella* use a T3S system to invade the colonic epithelium in humans and cause bacillary dysentery [5], [6]. The *S. flexneri* T3S system is composed of ∼50 genes, most of which are carried by a virulence plasmid [7]. Genes encoding components of the T3SA, translocators, chaperones, four effectors and the transcription activators VirB and MxiE are clustered in a 30-kb region, designated the entry region, of the virulence plasmid. Genes encoding 19 other effectors and the transcription activator VirF are scattered on the virulence plasmid and five to seven genes encoding effectors of the IpaH family are carried by the chromosome [8], [9]. At 37°C, genes of the entry region and a set of effector-encoding genes are expressed under the control of VirF and VirB [10], [11], leading to the assembly of the T3SA in the bacterial envelope [12]. The T3SA is weakly active when bacteria are growing in broth; it is activated upon contact of bacteria with host cells or exposure to the dye Congo red [13], [14] and is deregulated, *i.e.* constitutively active, in *ipaB* and *ipaD* mutants [15], [16]. Activation and deregulation of the T3SA specifically increase transcription of a set of effector-encoding genes carried by both the virulence plasmid and the chromosome [16]–[18].

Transcription of genes in response to the activation of the T3SA is dependent upon MxiE and IpgC that are produced independently of the TS3A activity [16]. MxiE is a transcription activator of the AraC family and IpgC is the chaperone for the translocators IpaB and IpaC that is required as a co-activator for MxiE [16], [19]–[21]. When the T3SA is not active, MxiE is associated with the T3SA substrate OspD1 acting as an anti-activator and IpgC is associated with IpaB and IpaC [21], [22]. Upon activation of the T3SA, the transit of OspD1, IpaB and IpaC releases MxiE and IpgC in the cytoplasm and a complex composed of MxiE and IpgC is proposed to activate transcription of target promoters [16], [22]. MxiE and IpgC are sufficient to activate transcription of target promoters in the absence of other virulence plasmid-encoded factors [16], [20]. Association of MxiE and IpgC has been confirmed upon co-expression of both proteins in *Escherichia coli* [23], however, binding of MxiE or the MxiE:IpgC complex to the DNA has not yet been demonstrated.

**Figure 1:**
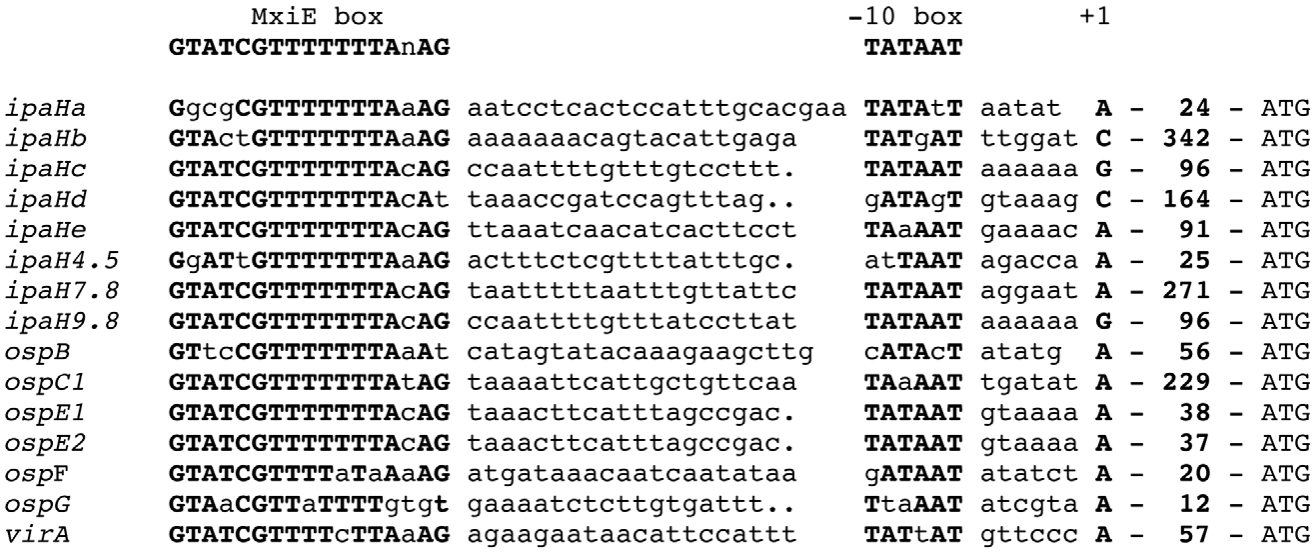
MxiE box-containing regions located 5′ of genes controlled by the T3SA activity. Sequences located 5′ to the translation start site (ATG) of MxiE-regulated genes are aligned with respect to the MxiE box and proposed −10 box and transcription start site (+1). Nucleotides identical to those present in the consensus sequence of the MxiE and −10 boxes (top row) are shown in bold uppercase characters. The number of nucleotides present in the 5′ UTR (from the nucleotide in position +1 to the nucleotide 5′ of the translation start site) is indicated.

Genes under the control of MxiE include the virulence plasmid genes *ospB*, *ospC1*, *ospD2*, *ospD3*, *ospE1*, *ospE2*, *ospF*, *ospG*, *virA*, *ipaH1.4*, *ipaH4.5*, *ipaH7.8* and *ipaH9.8* and some chromosomal *ipaH* genes [16]–[18], [22], [24]. Macroarray analysis indicated that the increased transcription of virulence plasmid genes observed in an *ipaB* mutant was abolished in an *ipaB mxiE* mutant [18]. A conserved 17-bp region, designated the MxiE box, was detected 33 bp upstream from the position corresponding to the 5′ extremity of *ospC1*, *virA* and *ipaH9.8* mRNA [25]. MxiE boxes exhibiting up to three differences with the MxiE box consensus sequence were also detected 5′ of *ospB*, *ospE1*, *ospE2*, *ospF*, *ipaH4.5*, *ipaH7.8* and the five different *ipaH* genes carried by the chromosome [18]. A MxiE box exhibiting five differences as compared to the consensus sequence is also present 43 bp 5′ of *ospG* (Fig. 1), the transcription of which is controlled by the T3SA activity [18], [26]. To investigate whether all these MxiE box-containing regions are endowed with a promoter activity and to compare these activities, similar DNA fragments comprising the MxiE and putative −10 boxes of 14 MxiE-regulated genes were cloned 5′ of *lacZ* in a promoter probe vector; recombinant plasmids were introduced into the *S. flexneri* wild-type strain, in which the T3SA is weakly active during growth in broth, and an *ipaB* mutant, in which the T3SA is constitutively active and MxiE-regulated genes are transcribed. Except for the MxiE box-containing region 5′ of *ospG*, all these ∼67-bp DNA fragments were endowed with a promoter activity that was regulated by the T3SA activity and the relative strengths of these MxiE-regulated promoters covered an eight-fold range. To investigate the potential effect of differences among MxiE boxes on the activity of these promoters, each MxiE box was introduced into the *ipaH9.8* promoter and, to assess the role of conserved nucleotides in MxiE boxes, each of these nucleotides was individually mutated in the *ipaH9.8* promoter. All natural MxiE boxes, except the one located 5′ of *ospG*, were found to be equally efficient in promoter activation and all nucleotides strictly conserved among MxiE boxes were found to play a role in, and some to be essential for, promoter activation. We also present evidence that the 5′ untranslated regions (UTR) of four MxiE-regulated genes enhance expression of the downstream gene and the 5′ UTRs of two other genes contain an ancillary promoter.

## Materials and Methods

### Bacterial strains and growth media

*S. flexneri* strains are derivatives of M90T-Sm (wild-type) [27], SF1076 (*ipaB4*), SF1070 (*ipaB4 mxiE2*) [16] and BS176 (pVir-, virulence plasmid-cured) [28]. Bacteria were grown in tryptic soy (TCS) broth. Ampicillin was used at 100 µg mL^−1^.

### Plasmids

DNA analysis, polymerase chain reactions (PCR), plasmid constructions and transformations of *E. coli* and *S. flexneri* strains were performed according to standard methods. DNA fragments were amplified from the virulence plasmid and the chromosome of the *S. flexneri* strain M90T-Sm (serotype 2a) using primers derived from the sequence of the virulence plasmid pWR100 of M90T [7] and the genome of the *S. flexneri* strain 8401 (serotype 5b) [29], respectively. Sequences of promoter regions and 5′ UTRs of chromosomal *ipaH* genes from M90T were identical to those of 8401. There are five different chromosomal *ipaH* genes, some of which are duplicated in different strains; the same genes are annotated with different names in different genomes and, for the sake of clarity, we used the letter-based nomenclature [18] in which duplicated *ipaH* genes are designated by the same letter (Table 1). The promoter probe vector pQF50 contains, in particular, two Rho-independent transcription termination sites followed by restriction sites for NcoI, BamHI, KpnI and HindIII, a ribosome binding site and the *lacZ* gene [30]. The KpnI and HindIII sites correspond to positions −44 to −39 and −26 to −21, respectively, with respect to the *lacZ* translation start site. Inserts carried by all plasmids constructed in this study were verified by sequencing.

**Table 1:** Annotations of chromosomal *ipaH* genes in *Shigella* genomes^a^.

Gene	Size (bp)	*S. flexneri*	*S. flexneri*	*S. flexneri*	*S. dysent.*	*S. sonnei*	*S. boydii*
		8401	301	2457T	197	046	227
*ipaHa1*	*1764*	*ipaH7*	*ipaH1*	*ipaH1* (fs)	*ipaH1*	*ipaH1*	*ipaH6*
*ipaHa2*	*1764*	*-*	*-*	*-*	*ipaH2* (IS)	*-*	*-*
*ipaHb1*	*1827*	*ipaH1*	*ipaH2*( fs)	*ipaH2*	*ipaH6*	*ipaH5*	*ipaH1*
*ipaHb2*	*1827*	*ipaH6*	*ipaH7*	*ipaH7*	*-*	*-*	*-*
*ipaHc*	*1716*	*ipaH2*	*ipaH3*	*-*	*ipaH4*	*ipaH3*	*ipaH4*
*ipaHd*	*1752*	*ipaH3*	*ipaH4*	*ipaH4*	*ipaH5*	*ipaH2*	*ipaH5*
*ipaHe1*	*1644*	*ipaH4*	*ipaH5* (IS*)*	*ipaH5*	*ipaH3* (IS*)*	*ipaH4*	*ipaH2*
*ipaHe2*	*1644*	*ipaH5*	*ipaH6* (IS)	*ipaH6* (fs)	*-*	*-*	*ipaH3*

a Names used to annotate the chromosomal *ipaH* genes in different genomes are indicated; in this study, we used the letter-based nomenclature indicated in the left column. Genes inactivated by a frameshift mutation or an insertion sequence are indicated (fs) and (IS), respectively, and genes absent from a genome are indicated by a hyphen.

**Figure 2:**
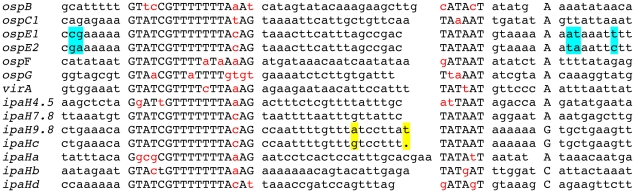
Minimal promoter regions cloned into pQF50. Sequences are aligned with respect to the MxiE and −10 boxes; nucleotides matching the consensus sequence of the MxiE box and the proposed −10 box are shown in uppercase characters. Nucleotides differing from the MxiE box and −10 box consensus sequences are shown in red. Nucleotides differing between the *ospE1* and *ospE2* promoter regions are highlighted in blue and nucleotides differing between the *ipaH9.8* and *ipaHc* promoter regions are highlighted in yellow. Each of these sequences was inserted between the NcoI (in 5′) and KpnI (in 3′) sites of pQF50.

For the analysis of minimal promoter regions, DNA fragments extending from 8 bp 5′ of the MxiE box to 11 bp 3′ of the proposed transcription start site of 14 MxiE-regulated genes were inserted between the NcoI and KpnI sites of pQF50 (Fig. 2). The fragment carrying the *ipaH9.8* promoter was also inserted between the NcoI and HindIII sites of pQF50.

For the analysis of specific nucleotides within the MxiE box, the region extending from positions −16 to +85 of the *ipaH9.8* promoter was first inserted between the BamHI and HindIII sites of pQF50 to construct pCB23. Double-stranded oligonucleotides were then inserted between the NcoI and BamHI sites of pCB23 to reconstruct a complete *ipaH9.8* promoter region and its 5′ UTR (from −57 to +85) inserted between the NcoI and HindIII sites of pQF50 and carrying a BamHI site between the MxiE and −10 boxes. The presence of the sequence 5′-GGATCC-3′ (BamHI) instead of 5′-TTATCC-3′ in positions −22 to −17 did not affect regulation of the promoter. Double-stranded oligonucleotides carrying variations or mutations in the MxiE box were inserted between the NcoI and BamHI sites of pCB23 to construct plasmids carrying a reconstituted *ipaH9.8* promoter (from −57 to +85) harboring different MxiE boxes.

For the analysis of the promoter activity of 5′ UTRs, DNA fragments encompassing the region located between position +2, with respect to the transcription start site of each promoter, and position −13, with respect to the translation start site of each gene, were inserted between the BamHI and HindIII sites of pQF50.

For the analysis of the role of 5′ UTRs on expression of *lacZ* transcribed from the *lac* promoter, a DNA fragment corresponding to nucleotides −39 to +1 of the *lac* promoter (5′-TTATTTGCTTTGTGAGCGGATAACAATTATAATAGATTCA-3′) was first inserted between the NcoI and BamHI sites of pQF50 to construct pCB55. DNA fragments encompassing the region encoding the 5′ UTR (as defined above) of each gene were then inserted between the BamHI and HindIII sites of pCB55, thereby placing each 5′ UTR between the *lac* promoter and *lacZ*. Coordinates of the 3′ end of the cloned 5′ UTR with respect to the original promoter are: *ipaH7.8*, +260; *ipaH9.8*, +85; *ipaHb*, +331; *ipaHc*, +85; *ipaHd*, +153; *ipaHe*, +74; *ospB*, +45; *ospC1*, +218; *virA*, +46. In the 5′ UTR of *ipaHe*, the C at position +70 was changed to a G to prevent pairing of the 3′ region of the 5′ UTR with the ribosome binding site of *lacZ*.

For the deletion analysis of the 3′ part of the *ipaH9.8* 5′ UTR, DNA fragments were inserted between the BamHI and HindIII sites of pCB24 to construct plasmids carrying an *ipaH9.8* promoter and various portions of the *ipaH9.8* 5′ UTR.

### β-galactosidase assay

The β-galactosidase activity present in bacteria grown for 16 h at 37°C in TCS medium was assayed by using the substrate *o*-nitrophenyl-β-D-galactopyranoside as described [31]. β-galactosidase activity is expressed in Miller units; values are the mean of at least three independent experiments performed in duplicate.

### mRNA folding prediction

Prediction of potential secondary structures of 5′ UTRs were performed with the program mfold [32] using the default parameters, as available at http://mobyle.pasteur.fr/cgi-bin/portal.py#forms::mfold.

## Results

### Relative strengths of MxiE box-containing promoters

**Figure 3:**
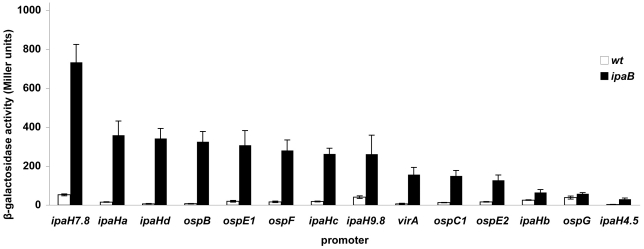
Promoter activity of MxiE box-containing regions. β-galactosidase activity was assayed in derivatives of the wild-type (open bars) and *ipaB* (filled bars) strains harboring a plasmid carrying a ∼67-bp fragment, encompassing nucleotides −57 to +11 with respect to the proposed transcription start site of each gene (Fig. 2), inserted 5′ of *lacZ* in the promoter probe vector pQF50. Gene names are indicated below the bars. Activities are expressed in Miller units.

Transcriptional fusions to some MxiE-regulated genes constructed previously carried DNA fragments of different sizes and varying in their respective 5′ and 3′ ends [25]. To confirm that all DNA regions containing a MxiE box carry a promoter and to compare the relative strengths of these promoters, similar DNA fragments encompassing the region extending from 8 bp 5′ of the MxiE box to 11 bp 3′ of the proposed transcription start site of each potential promoter were cloned upstream from *lacZ* in the promoter-probe vector pQF50 [30]. These fragments are 66 to 69 bp in length, depending on the size of the region located between the MxiE and −10 boxes (Fig. 2). Among the 15 different potential promoter regions, *i.e.* not taking into account duplicated copies of chromosomal *ipaH* genes (Table 1), only the fragment encompassing the MxiE box of *ipaHe* was not obtained in pQF50. Recombinant plasmids were introduced into the *S. flexneri* strains M90T-Sm (wt) and SF1076 (*ipaB4*); the T3SA is constitutively active in *ipaB* mutants, leading to the increased transcription of MxiE-regulated genes in these mutants as compared to the wild-type strain [16]. Derivatives of the wild-type strain contained five to 40 Miller units of β-galactosidase activity and derivatives of the *ipaB* strain contained increased activities, from 50 to 700 Miller units, except for the strain harboring the plasmid carrying the MxiE box-containing region of *ospG* (Fig. 3). In the later case, the low β-galactosidase activities detected in both the wild-type (38 Miller units) and the *ipaB* (58 Miller units) strains suggest that the MxiE box containing region 5′ of *ospG* does not contain a promoter regulated by the T3SA activity, consistent with previous observations made with a plasmid carrying a fragment encompassing 230 bp 5′ and 100 bp 3′ of the *ospG* translation start site [25]. These results confirmed that, in most cases, the ∼67-bp DNA fragment encompassing the MxiE and −10 boxes carries a promoter the activity of which is regulated by the T3SA activity. Differences in β-galactosidase activities expressed by the *ipaB* strain harboring each plasmid suggest that the relative strengths of MxiE-regulated promoters cover an eight-fold range, with *ipaH7.8*>*ospB*, *ospE1*, *ospF*, *ipaH9.8*, *ipaHa*, *ipaHd*, *ipaHc*>*virA*, *ospC1*, *ospE2*>*ipaH4.5*, *ipaHb*.

**Figure 4:**
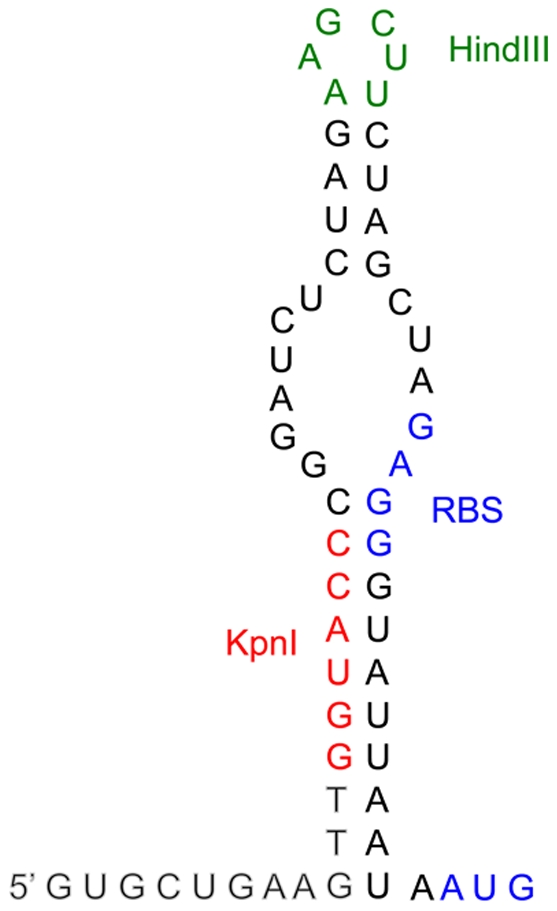
Potential secondary structure involving the sequence encoded by the KpnI-HindIII fragment of pQF50 and the *lacZ* ribosome binding site. The potential secondary structure of the 5′ UTR mRNA arising from the minimal *ipaH9.8* promoter cloned at the KpnI site of pQF50 is presented, with nucleotides specific of *ipaH9.8* shown in grey, the KpnI site in red, the HindIII site in green and the ribosome-binding site (RBS) and translation start codon of *lacZ* in blue characters.

β-galactosidase activities expressed by *ipaB* strains harboring plasmids carrying these minimal promoter regions inserted at the KpnI site of pQF50 were lower than the ones obtained previously with derivatives of pQF50 carrying 300 to 500-bp fragments encompassing the MxiE box inserted at the HindIII site (located 12 bp downstream from the KpnI site) [25]. Closer examination of the sequence of pQF50 revealed that the sequence of the KpnI-HindIII fragment is complementary to a part of the *lacZ* ribosome-binding site (Fig. 4), suggesting that a secondary structure of the mRNA might decrease translation of *lacZ* transcribed from plasmids carrying this KpnI-HindIII fragment. In the case of the cloned *ipaH9.8* promoter, the mRNA secondary structure masking the *lacZ* ribosome binding site involves three additional nucleotides 5′ of the KpnI site (Fig. 4). To test this hypothesis, the minimal promoter region of *ipaH9.8* was also inserted at the HindIII site of pQF50. In contrast to the wild-type and *ipaB* strains harboring the plasmid carrying the fragment cloned at the KpnI site that contained 40 and 260 Miller units of β-galactosidase activity, respectively, the wild-type and *ipaB* strains harboring the plasmid carrying the fragment cloned at the HindIII site contained 340 and 1,700 Miller units of β-galactosidase activity, respectively. These results indicated that the presence of the KpnI-HindIII fragment is responsible for a six-fold decrease in *lacZ* translation and subsequent constructions were made at the HindIII site of pQF50.

### Variable and conserved nucleotides in MxiE boxes

**Figure 5:**
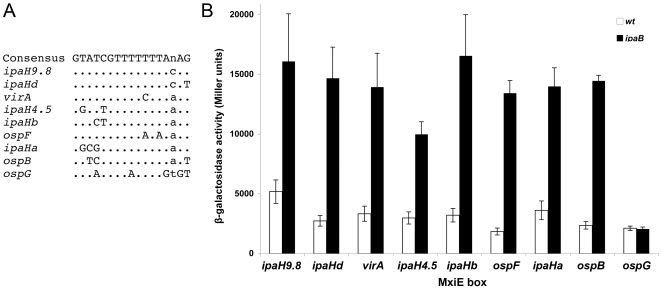
Effects of variations in the MxiE box on the *ipaH9.8* promoter activity. A: the MxiE box consensus sequence is shown on the upper row; only nucleotides differing from the consensus sequence in various MxiE boxes are indicated below, together with the name of the gene controlled by this MxiE box. Nucleotides corresponding to position 15 (indicated as n in the consensus sequence) are indicated in lower cases. MxiE boxes of *ipaHc*, *ipaHe*, *ipaH7.8*, *ospE1* and *ospE2* (being identical to the one of *ipaH9.8*) and the MxiE box of *ospC1* (differing from that of *ipaH9.8* only at position 15) are not shown. B: β-galactosidase activities were assayed in derivatives of the wild-type (open bars) and *ipaB* (filled bars) strains harboring a plasmid carrying the *ipaH9.8* promoter (from −57 to +85) with the MxiE box of genes indicated below the bars. Activities are expressed in Miller units.

All MxiE-regulated promoters tested in this study do not carry the same MxiE box (Fig. 1). To investigate the potential effect of variations in MxiE boxes on the promoter activity, each MxiE box was introduced into the *ipaH9.8* promoter cloned with its 5′ UTR (from −57 to +85) in pQF50 (Fig. 5A). For each plasmid, except that carrying the MxiE box of *ospG*, increased β-galactosidase activities were detected in the *ipaB* strain as compared to the wild-type strain (Fig. 5B), indicating that each MxiE box (except that of *ospG*) is functional in the context of the *ipaH9.8* promoter. Furthermore, β-galactosidase activities detected in the *ipaB* strain harboring plasmids carrying different MxiE boxes (other than that of *ospG*) were in the same range, from 10,000 to 16,000 Miller units, suggesting that all these MxiE boxes have the same efficiency for the MxiE-dependent activation of the *ipaH9.8* promoter. The lack of activity of the *ipaH9.8* promoter carrying the *ospG* MxiE box suggests that this box, which exhibits five base substitutions as compared to the consensus sequence, is not functional; this result is consistent with the weak and unregulated promoter activity observed for the MxiE box-containing region of *ospG* (Fig. 3). The *ipaH9.8* promoter carrying the *ipaH4.5* and *ipaHb* MxiE boxes exhibited high activity in the *ipaB* strain, indicating that these MxiE boxes are functional; accordingly, the weak activity observed for the *ipaH4.5* and *ipaHb* promoters (Fig. 3) might not be due to mutations in their MxiE boxes.

**Figure 6:**
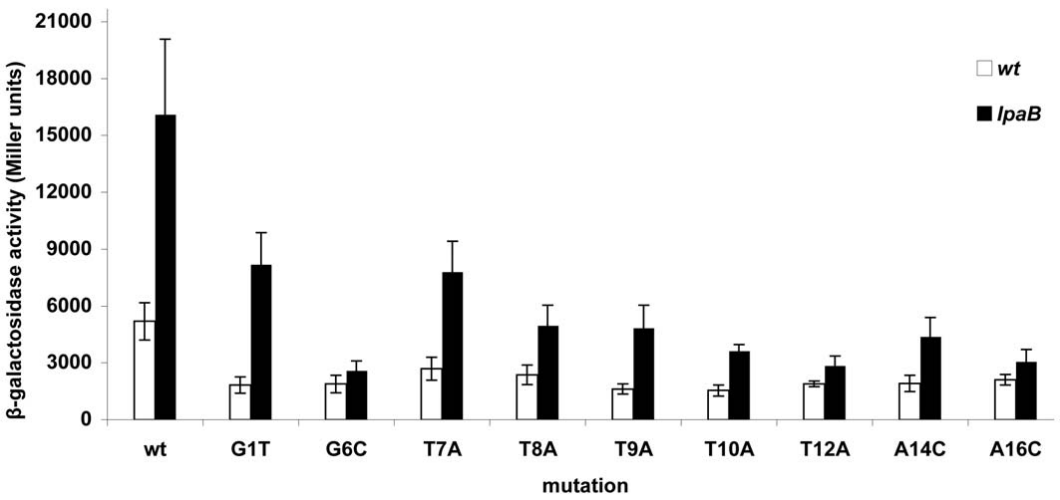
Effects of mutations in conserved positions of the MxiE box on the *ipaH9.8* promoter activity. β-galactosidase activity was assayed in derivatives of the wild-type (open bars) and *ipaB* (filled bars) strains harboring a plasmid carrying the *ipaH9.8* promoter (from −57 to +85) with the mutation in the MxiE box indicated below the bars. Activities are expressed in Miller units.

Results presented above indicated that, with the exception of the *ospG* MxiE box, variations in MxiE boxes do not affect the activity of the *ipaH9.8* promoter in the *ipaB* strain. Nine out of 17 nucleotides are strictly conserved among functional MxiE boxes, including a G at positions 1 and 6, a T at positions 7, 8, 9, 10 and 12 and an A at positions 14 and 16 (Fig. 1). To determine the importance of these conserved positions on the activity of MxiE-regulated promoters, these nucleotides were mutated individually in the *ipaH9.8* promoter (from −57 to +85) and β-galactosidase activity was assayed in derivatives of the wild-type and *ipaB* strains harboring each plasmid. Mutations G1T and T7A led to a two-fold decrease in β-galactosidase activity, mutations T8A, T9A and A14C to a three-fold decrease and mutations G6C, T10A, T12A and A16C to a six-fold decrease, abolishing almost completely the activity of the promoter in the *ipaB* mutant (Fig. 6). These results indicated that all nucleotides strictly conserved in MxiE-boxes examined in this study are essential for the full activity the *ipaH9.8* promoter when the T3SA is active.

**Figure 7:**
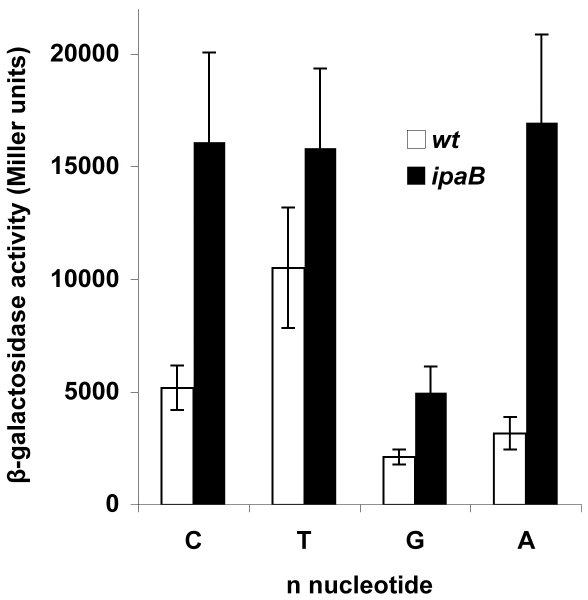
Effects of the nucleotide in position 15 of the MxiE box on the *ipaH9.8* promoter activity. β-galactosidase activity was assayed in derivatives of the wild-type (open bars) and *ipaB* (filled bars) strains harboring a plasmid carrying the *ipaH9.8* promoter (from −57 to +85) with the nucleotide indicated below the bars in position 15 of the MxiE box. Activities are expressed in Miller units.

Among functional MxiE boxes, *i.e.* not including that of *ospG*, position 15 is occupied by a C (seven occurrences), an A (six occurrences) or a T (one occurrence) (Fig. 1). To investigate the importance of the nature of the nucleotide in position 15 on the activity of the promoter, C15 in the *ipaH9.8* promoter (from −57 to +85) was changed to A, G or T. The C15A mutation had no effects on the promoter activity in the wild-type and *ipaB* strains, whereas the C15G mutation drastically reduced the promoter activity in the *ipaB* strain (Fig. 7). Unexpectedly, the C15T mutation led to a high activity of the *ipaH9.8* promoter in the wild-type strain, although no effect was observed in the *ipaB* strain (Fig. 7). With the plasmid carrying the C15T mutation, similar β-galactosidase activities were detected in the wild-type and *ipaB mxiE* strains, confirming that the high activity detected in the wild-type strain was not dependent upon MxiE (data not shown). The increased activity observed upon introducing a T at position 15 in the *ipaH9.8* promoter might be due to the creation of an artificial −35 region. Overall, these results on the effect of the nucleotide at position 15 on the activity of the *ipaH9.8* promoter are consistent with the frequent occurrence of a C and an A and the exclusion of a G and a T (with the exception of the MxiE box of *ospC1*) at this position in MxiE boxes.

### Analysis of the 5′ UTR of MxiE-regulated genes

The *ipaB* strains harboring plasmids carrying the *ipaH9.8* promoter either alone (from −57 to +11) or together with its 5′ UTR (from −57 to +85) inserted 5′ of the HindIII site in pQF50 contained 1,700 and 15,000 Miller units of β-galactosidase activity, respectively. This observation led us to investigate the role of the 5′ UTR of *ipaH9.8*. The increased β-galactosidase activity observed with the plasmid carrying the 5′ UTR could potentially be due to either an ancillary promoter carried by the DNA region corresponding to the 5′ UTR or an effect of the 5′ UTR mRNA on expression of the downstream gene. The study of the *ipaH9.8* 5′ UTR was extended to the 5′ UTRs of other MxiE-regulated genes displaying a wide heterogeneity in length (Fig. 1).

**Figure 8:**
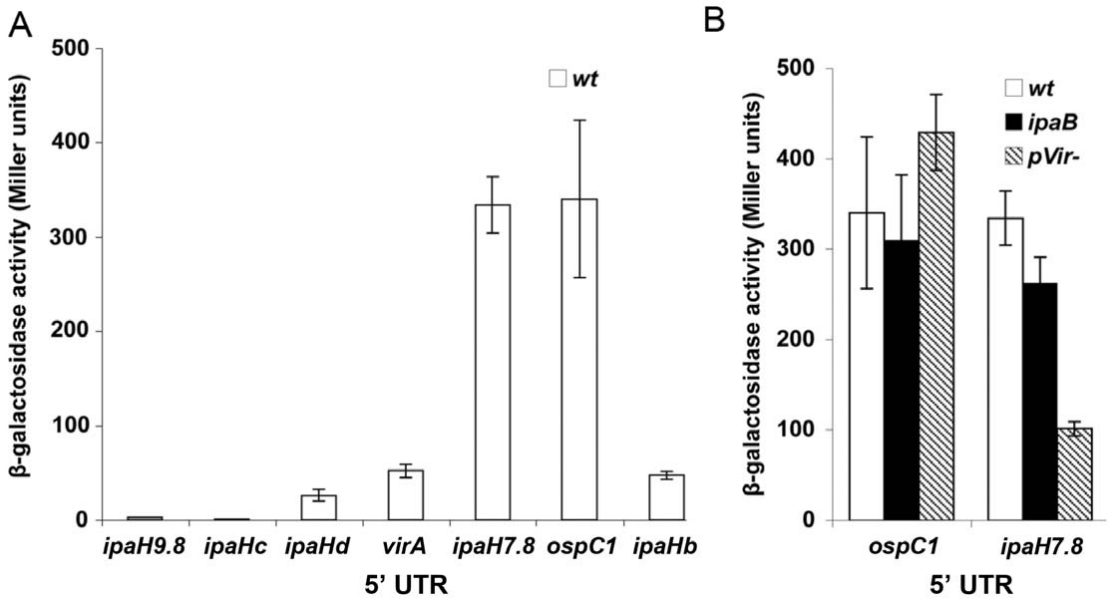
Promoter activity of the 5′ UTR of MxiE-regulated genes. A: β-galactosidase activity was assayed in derivatives of the wild-type (open bars) strain harboring plasmids carrying the 5′ UTR of genes indicated below the bars inserted 5′ of *lacZ* in the promoter probe vector pQF50. The cloned 5′ UTRs extend from position +2 (with respect to the transcription start site) to position −13 (with respect to the translation start site). B: β-galactosidase activity was assayed in derivatives of the wild-type (open bars), *ipaB* (filled bars) and virulence plasmid-cured (pVir-, dashed bars) strains harboring derivatives of pQF50 carrying the 5′ UTR of genes indicated below the bars. Activities are expressed in Miller units.

To determine whether the regions encoding the various 5′ UTRs might carry a promoter, DNA fragments corresponding to 5′ UTRs longer than 50 nucleotides were inserted 5′ of *lacZ* in pQF50. To avoid potential interferences between the translation start site of the MxiE-regulated gene and the translation start site of *lacZ*, the cloned fragment did not include the last 12 bp encoding the 3′ extremity of the original 5′ UTR. The derivative of the wild-type strain harboring the vector pQF50, *i.e.* without any fragment cloned 5′ of *lacZ*, contained less than 10 Miller units of β-galactosidase activity. Weak (<50 Miller units) β-galactosidase activities were detected in the wild-type strain harboring plasmids carrying the *ipaHb*, *ipaHc*, *ipaHd*, *ipaH9.8* and *virA* 5′ UTRs, suggesting that these regions do not contain an active promoter (Fig. 8A). In contrast, strains harboring plasmids carrying the *ipaH7.8* and *ospC1* 5′ UTRs contained ∼300 Miller units of β-galactosidase activity, indicating that each of these 5′ UTRs is likely endowed with a promoter; for both plasmids, β-galactosidase activities detected in the *ipaB* strain were similar to those detected in the wild-type strain (Fig. 8B), indicating that these promoters are not regulated by the T3SA activity. For the *ospC1* 5′ UTR, similar activities were detected in the virulence plasmid-cured and wild-type strains (Fig. 8B), indicating that the promoter carried by the *ospC1* 5′ UTR is not controlled by a virulence plasmid-encoded factor. In contrast, for the *ipaH7.8* 5′ UTR, a two-fold reduction in the β-galactosidase activity was observed in the virulence plasmid-cured strain as compared to the wild-type strain (Fig. 8B). These results suggested that the promoter carried by the *ipaH7.8* 5′ UTR is regulated, in part, by an unknown virulence plasmid-encoded factor.

**Figure 9:**
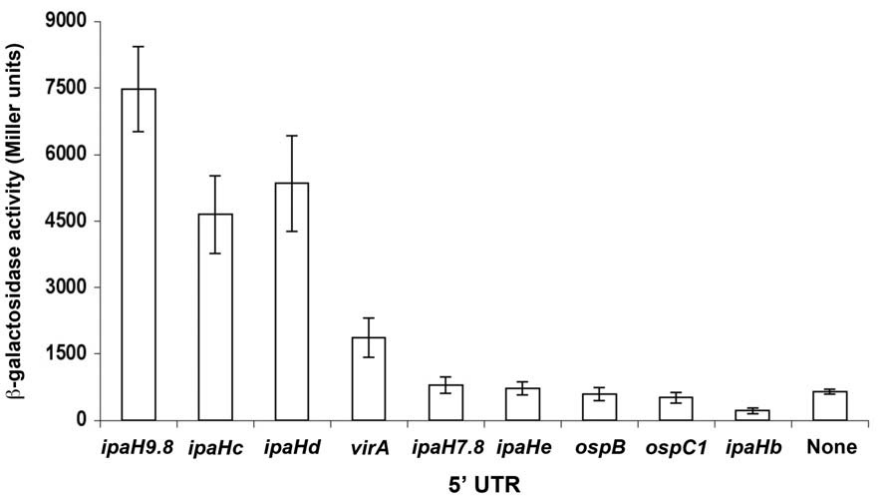
Expression of *lacZ* from plasmids carrying 5′ UTRs inserted 3′ of the *lac* promoter. β-galactosidase activity was assayed in derivatives of the wild-type strain harboring plasmids carrying the 5′ UTR of genes indicated below the bars inserted between the *lac* promoter and *lacZ*. The cloned 5′ UTRs extend from position +2 (with respect to the transcription start site) to position −13 (with respect to the translation start site) of genes indicated below the bars. Activities are expressed in Miller units.

To investigate the potential role of the 5′ UTR mRNA on expression of the downstream gene, we placed the 5′ UTRs of MxiE-regulated genes under the control of a different promoter. A minimal *lac* promoter (from −39 to +1) was first cloned 5′ of *lacZ* in pQF50 and DNA fragments encoding the 5′ UTRs were inserted between this *lac* promoter and *lacZ*. The *lac* promoter is constitutively active in *S. flexneri*, due to the absence of the *lacI* gene. The wild-type strain harboring the plasmid carrying the *lac* promoter alone contained approximately 600 Miller units of β-galactosidase activity and this activity was not increased upon insertion of the 5′ UTR of *ipaH7.8*, *ipaHb*, *ipaHe*, *ospB* and *ospC1* (Fig. 9). In contrast, increased β-galactosidase activities (from 1,900 to 7,500 Miller units) were detected in the wild-type strain harboring plasmids carrying the 5′UTR of *virA*, *ipaHd*, *ipaHc* and *ipaH9.8* (Fig. 9), even though these 5′ UTRs are not endowed with a promoter activity (Fig. 8A). As expected from the constitutive nature of the *lac* promoter in this context, similar activities were detected in both the wild-type and *ipaB* strains (data not shown), which also indicated that the increased expression of *lacZ* observed in the presence of these 5′ UTRs is not controlled by the T3SA activity.

**Figure 10:**
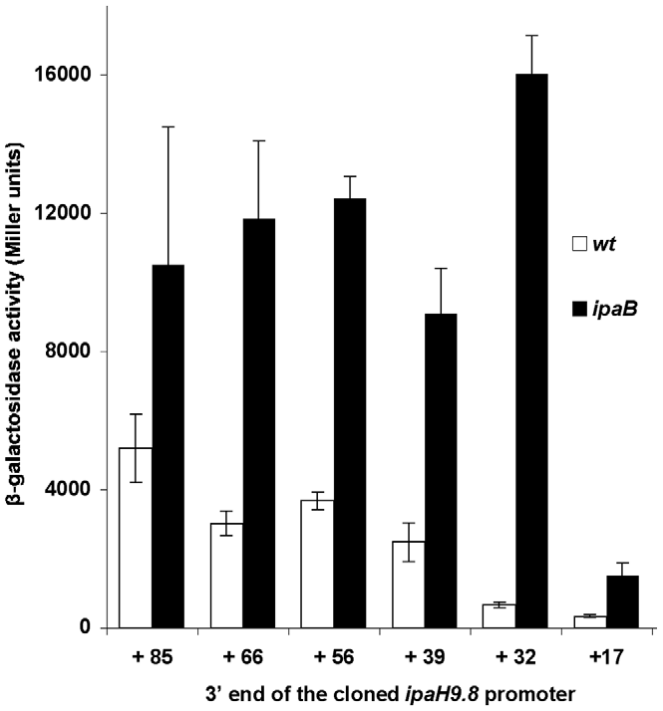
Expression of *lacZ* from plasmids carrying the *ipaH9.8* promoter with 5′ UTRs of different lengths. β-galactosidase activity was assayed in derivatives of the wild-type (open bars) and *ipaB* (filled bars) strains harboring plasmids carrying the *ipaH9.8* promoter with 5′ UTRs of different lengths. Numbers below the bars indicate the position of the 3′ end of the cloned 5′ UTR. Activities are expressed in Miller units.

**Figure 11:**
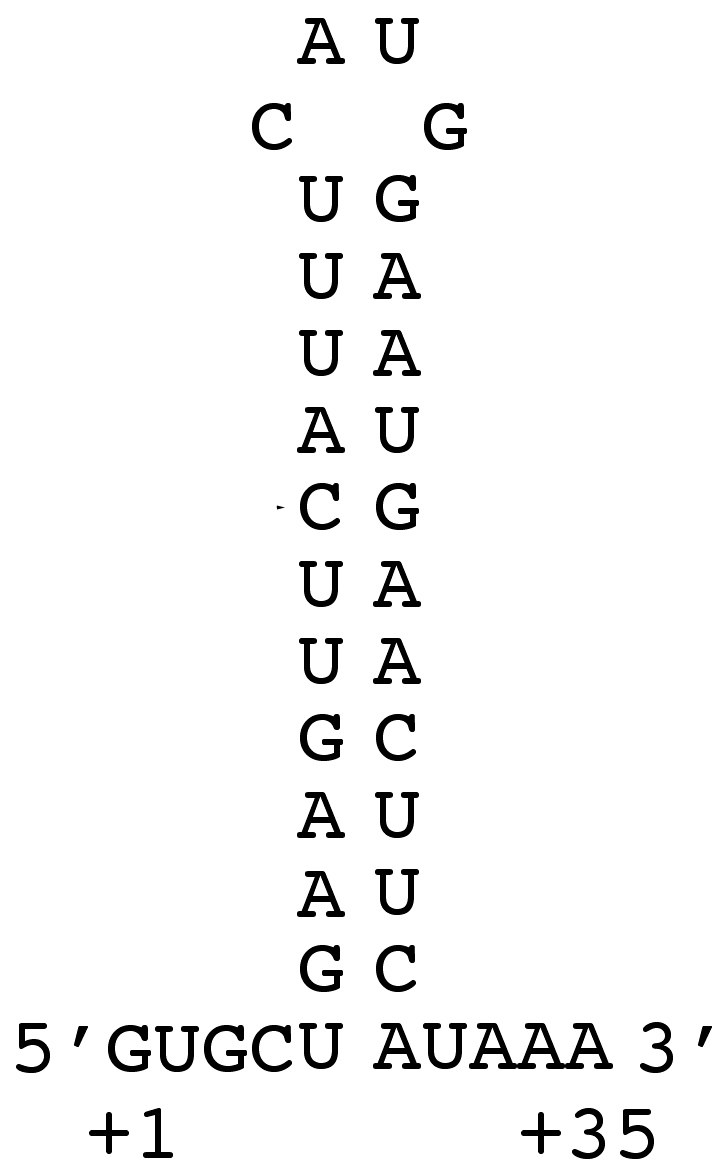
Potential secondary structures of the mRNA in the 5′ UTR of *ipaH9.8* and *ipaHc*.

To identify the region of the *ipaH9.8* 5′ UTR responsible for the increased expression of the reporter gene, we constructed plasmids carrying the *ipaH9.8* promoter with shortened versions of its 5′ UTR, by truncating the 5′ UTR from its 3′ extremity. These plasmids were introduced into the wild-type and *ipaB* strains. Derivatives of the *ipaB* strain harboring plasmids encoding a 5′ UTR ending at positions +85, +66, +56, +39 and +32 all produced high amounts of β-galactosidase (Fig. 10). Reducing the length of the 5′ UTR to 17 nucleotides led to a strong decrease in β-galactosidase activity. Analysis of the sequence spanning nucleotides 1 to 32 of the *ipaH9.8* 5′ UTR indicated that the corresponding mRNA might adopt a stem and loop structure, with base pairing between nucleotides +5 to +16 and +21 to +32 (Fig. 11). These results suggested that a secondary structure formed at the 5′ extremity of the *ipaH9.8* 5′ UTR, as well as the *ipaHc* 5′ UTR containing the same sequence at the same place, is involved in the increased expression of the reporter gene.

## Discussion

We used an analytical approach to characterize the *cis*-acting elements involved in expression of genes controlled by the T3SA activity in *S. flexneri*, separating the promoter regions from their associated 5′ UTR and investigating the role of variable and conserved nucleotides among their MxiE boxes. The choice of the DNA fragment carrying the minimal promoter region of MxiE-regulated genes was based on the previously determined 5′ extremity of *ospC1*, *ipaH9.8* and *virA* mRNA [25]. DNA fragments extending from 8 bp 5′ of the MxiE box to 11 bp 3′ of the proposed transcription start site were shown here to carry promoters regulated by the T3SA activity, except for *ospG*. Although the mRNA 5′ extremity of all MxiE-regulated genes has not been determined, the regulated expression of *lacZ* from regions restricted to the MxiE box and proposed −10 box and transcription start site is evidence that these regions contain promoters controlled by the T3SA activity, but does not rule out that additional promoters might be present 5′ of these MxiE-regulated genes.

β-galactosidase activities detected in the *ipaB* strain harboring plasmids carrying the minimal promoter regions of MxiE-regulated genes suggest that their relative strengths are: *ipaH7.8*>*ospB*, *ospE1*, *ospF*, *ipaH9.8*, *ipaHa*, *ipaHd*, *ipaHc*>*virA*, *ospC1*, *ospE2*>*ipaH4.5*, *ipaHb*. These differences in promoter strengths do not appear to be caused by differences in their MxiE box. Indeed, promoters carrying the same MxiE box, such as the *ipaH7.8*, *ipaH9.8*, *ipaHc*, *ospE1* and *ospE2* promoters, have different strengths. Furthermore, natural MxiE boxes exhibiting up to three differences as compared to the consensus sequence, *i.e.* excluding that of *ospG*, were equally efficient for activation of the *ipaH9.8* promoter. Differences in promoter strengths do not appear to be due to differences in the spacing between the MxiE box and the −10 box because (i) the *ipaH9.8* and *ipaHc* promoters differ mostly by an insertion or a deletion of one bp 5′ of the −10 box and exhibit similar activities; (ii) deleting one bp 3′ of the *ipaH9.8* MxiE box did not affect the activity of the promoter (data not shown); (iii) and, among MxiE-regulated promoters tested, there are no correlations between the strength of the promoter and the size of the region located between the MxiE box and the −10 box. Thus, elements controlling the relative strength of each promoter remain to be identified. In the case of the almost identical *ospE1* and *ospE2* promoters, the lower activity of the *ospE2* promoter might be due to the presence of nucleotides TA at positions +2 and +3 in *ospE2* instead of AT in *ospE1*. The weak activity of the *ipaH4.5* and *ipaHb* promoters might be due to an altered −10 box in the *ipaH4.5* promoter and the presence of a C at the expected position for the transcription start site of the *ipaHb* promoter, which remains to be tested.

Only weak promoter activity was exhibited by the *ospG*, *ipaH4.5* and *ipaHb* MxiE box-containing regions and, in the case of *ospG*, this promoter activity was not regulated by the T3SA activity. The *ipaH4.5* and *ospG* genes are located downstream from *ipaH7.8* and *ipaH9.8*, respectively, and the MxiE-regulated expression of *ipaH4.5* and *ospG* [18], [26] is probably mostly dependent upon the upstream *ipaH7.8* and *ipaH9.8* promoters. No other MxiE boxes were detected upstream from *ipaHb*, suggesting that this gene is transcribed much less than other MxiE-regulated genes.

Among positions conserved between MxiE boxes, G6, T10, T12 and A16 appear to be essential for promoter activity. A decreased activity of the *ipaH9.8* promoter was also observed upon replacement of T8, T9 and A14 (three-fold decrease) and G1 and T7 (two-fold decrease). Thus, changes of any of the strictly conserved nucleotides among MxiE boxes decreased the promoter activity. There is a remarkable stretch of seven consecutive Ts (from T7 to T13) in the MxiE box consensus sequence and this stretch of Ts is conserved in 12 MxiE boxes (Fig. 1). However, the integrity of this stretch of Ts is not essential for the MxiE-dependent activity of the promoter. Indeed, the stretch is interrupted in the MxiE box of *ospF* (harboring T11A and T13A) and *virA* (harboring T11C), both of which were shown here to be as functional as MxiE boxes containing an uninterrupted stretch of Ts. Furthermore, introducing the mutations T11A or T13A in the *ipaH9.8* promoter did not affect its activity (data not shown). Similar β-galactosidase activities were detected when position 15 of the MxiE box of the *ipaH9.8* promoter was occupied by either a C or an A, consistent with the preferential occurrence of these nucleotides at this position in MxiE boxes. Introducing a G at position 15 abolished promoter activation, which is also consistent with the observation that a G is absent at this position in MxiE boxes. The C15T mutation increased the *ipaH9.8* promoter activity in a manner that was not dependent upon MxiE; because a T is present at position 15 in the *ospC1* MxiE box and the *ospC1* promoter did not exhibit a high activity in the wild-type strain, the increased basal activity of the *ipaH9.8* promoter carrying a T at position 15 is tentatively interpreted as the result of the creation of an artificial promoter −35 region.

The observation that the presence of the 5′ UTR of *ipaH9.8* induced to a marked increased in the production of β-galactosidase led us to investigate the potential role of the 5′ UTR of MxiE-regulated genes. The DNA regions encoding the *ospC1* and *ipaH7.8* 5′ UTRs were found to carry ancillary promoters that are not controlled by the T3SA activity. Comparison of β-galactosidase activities produced by bacteria harboring plasmids containing either the MxiE-regulated promoters or the 5′ UTR-carried promoters must take into account that MxiE-regulated promoters were inserted at the KpnI site whereas 5′ UTR were inserted at the HindIII site of pQF50. Indeed, in the course of this study, we found that the presence of the KpnI-HindIII fragment of pQF50 was responsible for a six-fold decrease in β-galactosidase production, presumably due to the formation of a secondary structure in the mRNA masking the *lacZ* ribosome-binding site. By using this six-fold factor to standardize β-galactosidase activities, calculated values for promoters carried by the *opsC1* and *ipaH7.8* 5′ UTRs cloned at the KpnI site are ∼50 Miller units (in both the wild-type and *ipaB* strains). This estimate is in the same range as the β-galactosidase activities detected for *ospC1* and *ipaH7.8* MxiE-regulated promoters in the wild-type strain and is much lower than the activities detected for these promoters in the *ipaB* strain (150 Miller units for the *opsC1* promoter and 750 Miller units for the *ipaH7.8* promoter). These considerations suggest that the activity of 5′ UTR-carried promoters is similar to the basal activity of MxiE-regulated promoters when the T3SA is not active; however, the contribution of these 5′ UTR-carried promoters to the overall transcription of *ospC1* and *ipaH7.8* is very weak once the T3SA is activated.

Insertion of the 5′ UTRs of *virA*, *ipaHd*, *ipaHc* and *ipaH9.8* between the *lac* promoter and *lacZ* led to three- to twelve-fold increases in β-galactosidase activity. For *ipaH9.8*, the region responsible for this effect was mapped to the first 32 nucleotides of the 5′ UTR that are predicted to fold into a stem and loop structure. The same sequence is present in the *ipaHc* 5′ UTR and is probably involved in the increased expression of *lacZ* also observed with this 5′ UTR. A stem and loop structure formed at the 5′ extremity of the *E. coli ompA* and *papA* 5′ UTRs was demonstrated to function as a potent mRNA stabilizer [33]–[35]. The presence of a secondary structure at or very near the mRNA 5′ end, rather than the sequence of the stem and loop, was shown to be crucial for mRNA stability [34]. Determinants involved in the increased expression of the reporter gene observed upon insertion of *virA* and *ipaHd* 5′ UTRs between the *lac* promoter and *lacZ* have not been characterized. Various potential secondary structures are predicted in the mRNA corresponding to the *ipaHd* 5′ UTR, however, they are not located exactly at the 5′ extremity of the 5′ UTR and many secondary structures are possible in the 46-nucleotide *virA* 5′ UTR containing 40 As and Ts. In *Listeria monocytogenes*, the *inlA*, *actA* and *hly* 5′ UTRs have been shown to have a positive effect on expression of downstream genes, although specific sequences or mRNA secondary structures responsible for this effect have not been identified [36]–[39].

In conclusion, expression of MxiE-regulated genes involves an array of *cis*-acting elements, including MxiE boxes of different sequences, promoters of different strengths and 5′ UTRs of different lengths and functions. Combination of these elements might permit the control of gene expression over a wider range. All natural MxiE boxes appear to have the same efficiency for activation of the *ipaH9.8* promoter, whereas mutations introduced in any of the positions conserved among MxiE boxes decreased the *ipaH9.8* promoter activity in the *ipaB* strain. The 5′ UTRs of *ipaH9.8*, *ipaHc* and *ipaHd* were found to increase expression of the downstream gene by an approximately ten-fold factor, most likely by functioning as mRNA stabilizers.
